# Efficient induction of comprehensive immune responses to control pathogenic E. coli by clay nano-adjuvant with the moderate size and surface charge

**DOI:** 10.1038/s41598-017-13570-2

**Published:** 2017-10-17

**Authors:** Weiyu Chen, Huali Zuo, Timothy J. Mahony, Bing Zhang, Barbara Rolfe, Zhi Ping Xu

**Affiliations:** 1 Australian Institute for Bioengineering and Nanotechnology, University of Queensland, St Lucia, QLD 4072 Australia; 2 Queensland Alliance for Agriculture and Food Innovation, The University of Queensland, St Lucia, QLD 4072 Australia; 3 Department of Agriculture and Fisheries, Brisbane City, QLD 4000 Australia

## Abstract

In recent decades, diseases caused by pathogenic *Escherichia coli* (*E. coli*), enterohaemorrhagic *E. coli* (EHEC) O26 have been increasingly reported worldwide, which are as severe as those caused by EHEC strain O157:H7 and require effective intervention strategies. Herein, we report the application of clay nanoparticles, i.e. hectorites as effective nano-adjuvants for vaccination against EHEC O26 colonization. We show that medium size HEC (hectorite, around 73~77 nm diameter) is able to induce efficient humoral and cellular immune responses against EHEC antigen - intimin β (IB), which are significantly higher than those triggered by commercially used adjuvants - QuilA and Alum. We also demonstrate that mice immunized with IB adjuvanted with HEC nanoparticles elicit sufficient secretion of mucosal IgA, capable of providing effective protection against EHEC O26 binding to ruminant and human cells. In addition, we demonstrate for the first time that hectorites are able to initiate maturation of RAW 264.7 macrophages, inducing expression of co-stimulatory cytokines at a low nanoparticle concentration (10 μg/mL). Together these data strongly suggest that hectorite with optimized size is a highly efficient vaccine nano-adjuvant.

## Introduction

Although the majority of *E. coli* are innocuous to human, EHEC strains are responsible for a broad-spectrum of diseases, including mild diarrhoea, haemorrhagic colitis and haemolytic uraemic syndrome^1,2^. As the major pathotype of *E. coli*, EHEC is able to infect victims furtively through food-borne transmission or direct contact with domesticated animals^3^. Through generation of attaching and effacing (A/E) lesion and verotoxins^4^, EHEC strains can trigger series of diseases in potential hosts and cause food poisoning outbreaks globally with varying severity depending on the virulence properties of the strain involved^5,6^.

Although the majority of illnesses are caused by EHEC O157:H7, the other serotypes are also important. In the United States alone, more than 37,000 illnesses are induced by non-O157:H7 EHEC serotypes annually^7^. Among them, the most common serotype, EHEC O26 has been associated with 22% of all these cases. EHEC O26 has been isolated from ruminant herds in Europe, Asia, Australia, North and South America, and is emerging as a major pathotype worldwide^8–10^. EHEC O26 is capable of causing severe diseases including haemolytic uremic syndrome (HUS), which are normally triggered by EHEC O157^11^. Even worse, O26 strains exert great pressure on animal husbandry by potentially causing illness to livestock after infection^12,13^.

Like most EHEC, EHEC O26 initiates adhesion to host cells via generation of intimin, a secreted protein (adhesin) encoded by the *eae* gene, which is critically essential for bacterial attachment and A/E lesion formation^14^. Different from the O157 strain that produces intimin-γ, O26 along with other serotypes is identified as a distinct phylogenetic group by secretion of intimin-β (IB)^15^. In previous studies, the application of antibodies against the C-terminal of IB has been shown to decrease bacterial adherence to mammalian cells, while immunization with the C-terminal of IB formulated with complete Freund’s adjuvant stimulated a strong humoral response against EHEC, indicating that the C-terminal of IB is an effective antigen and could contribute to improving the control of EHEC^16,17^.

With assistance from adjuvants such as Alum, QuilA and Cholera toxin B subunit (CTB)^18,19^, vaccination offers a remarkable and affordable public health strategy compared with other remedial treatments such as antimicrobials, monoclonal antibodies and probiotic therapies^20,21^. Moreover, the application of an effective vaccine could significantly reduce the emergence of the increasing number of antimicrobial resistant diarrheagenic *Escherichia coli* generated by the indiscriminate use of antibiotics, the most common therapy against bacterial infections^22^. However, side effects could result from vaccination in both human and animal, such as induced hemolytic activity and local inflammation at the injection site^23,24^. Even the FDA-approved and widely used adjuvant, Alum, has undesirable drawbacks, including local inflammation and preferential Th2-biased immune response and non-biodegradability, i.e. the adjuvant persists at the site of injection for more than one year^25,26^.

In recent decades, nanomaterials have emerged as candidates for next-generation adjuvants. However, the majority of nano-adjuvants induce immune responses that are inferior, or similar to, those generated by QuilA^19,27^. Comparatively, clay nanomaterials, such as hectorites (HEC), do not only meet the ideal prerequisites for a desirable adjuvant with safe, stable, biodegradable and affordable features^28^, but also offer efficient affinities for protein-based antigen molecules via electrostatic force^29^. Laponite, a synthetic hectorite, is a disk-shaped nanoparticle with a large surface area^29^, and generally regarded as safe (GRAS) by the FDA as a natural product that could be degraded into non-toxic products (Na^+^, SiO_3_^2−^, Mg^2+^, Li^+^)^30^. Once laponite nanoparticles are taken up by macrophage cells in the injection site, they may also undergone similar biodegradation processes in the late endosome and lysosome^31^. Jointly, these properties suggest that hectorite nanomaterial has considerable potential as effective nano-adjuvant.

As the key mediator during vaccine stimulation, macrophages, the primary antigen presenting cells (APCs) are of vital importance in promoting immune responses, facilitating both cellular and humoral responses^32^. By expressing co-stimulatory molecules and major histocompatibility complex (MHC) antigens including class I and II, macrophages can efficiently prime naive T cells^33^. Cytokines secreted from stimulated macrophages, including IL-6 and TNF-α, act synergistically to further augment the immune responses^34^. Since macrophages are one of first cells that come into contact with foreign bodies, the effective activation of macrophages by an adjuvant would strongly suggest its usefulness in stimulating potent immune responses. To the best of our knowledge, there are no reports regarding the activation effect of hectorite on macrophage cells.

In our previous study, the optimization of the nanoparticle size had shown a significant impact on the adjuvanticity of layer double hydroxide (LDH) nanoparticles^31^. Meanwhile, HEC (Laponite WXFP) also exhibited similar adjuvant efficiency to LDH in vaccine application^31^, but the optimal size and surface charge of hectorite nanoparticles for use as adjuvants has not been determined. In this paper, we report our investigation results about hectorite nanoparticles as nano-adjuvants in inducing various immune responses against the EHEC O26 antigen IB. Our results demonstrate that hectorite nanoparticles with the medium size and surface charge elicit strong cellular and humoral responses to IB, which are more valid than those induced by commercial adjuvants (Alum and QuilA) and more effectively protect host cells from invasion by EHEC O26. Collectively, these data indicate the potential of HEC nanomaterial as highly effective nano-adjuvants and antigen carriers for vaccine applications.

## Results

### Physicochemical features of clay nanoparticles

**Figure 1:**
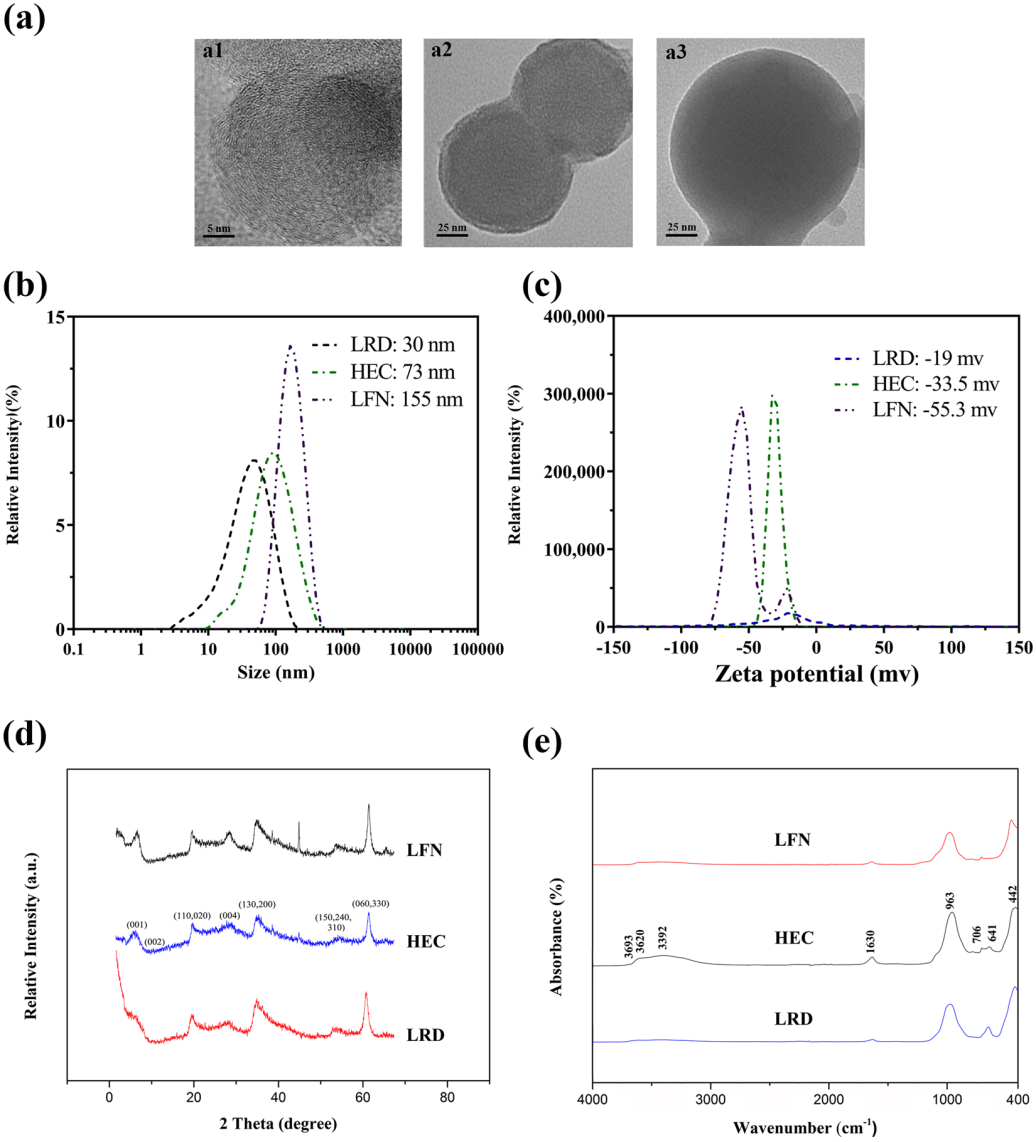
Physicochemical characterisation of different synthetic hectorite nanoparticles (HEC, LFN and LRD) investigated in this study. (**a**) Representative transmission electron microscope images of LRD (a1), HEC (a2) and LFN (a3); (**b**) Hydrodynamic sizes, (**c**) zeta potentials (**d**) XRD and (**e**) FTIR spectra of HEC, LFN and LRD nanoparticles.

As shown in Fig. 1 and Supplementary Fig. S1, the three hectorites, Laponite RD (LRD), Laponite WXFP (HEC) and Laponite FN (LFN) have the typical sheet-like morphology with lateral dimensions from 30 to 200 nm according to TEM images, which are uniformly sized except for LFN that has relatively boarder size range (from 50 to 200 nm). Consistently, the Z-average particle size of three hectorites was 30, 73 and 155 nm, respectively, with the polydispersity index (PDI) ranging from 0.163 to 0.397. These hectorite nanosheets carry negative charges, with the zeta potential of −55.3 ± 2.1, −33.5 ± 1.2 and −19.0 ± 3.1 mV for LFN, HEC and LRD, respectively (Fig. 1c and Table S1 in supplementary information). The average hydrodynamic size and the zeta potential of HEC nanoparticles were slightly different from our previous reports (77 nm and −41.1 mV)^31^. The X-ray diffraction (XRD) patterns are typical for hectorite, with the d-spacing (d_001_) being 1.49 nm (LRD), 1.42 nm (HEC) and 1.35 nm (LFN) (Fig. 1d)^35^, respectively, and in consistence with previous reports^36^. The typical infrared peaks, such as those at 3693 (ν_O-H_ in Mg-OH), 3620 (ν_O-H_ in Si-OH) and 963 (ν_Si-O_) and 442 cm^−1^ (ν_M-O_), were observed in the FTIR spectrum (Fig. 1e), identical to previous reports for hectorite materials^35^. Particles in alum hydroxide gel were aggregates, as shown in TEM image, and the average aggregate size was around 700 ~ 800 nm and the zeta potential 17.9 mV via DLS (supplementary Table S1 and Fig. S2).

### Effective adsorption of IB on clay nanoparticles

The interactions between the EHEC protein (IB) and clay nanoparticles were first indicated by the changes in zeta potential in aqueous suspension (Table S1, see supplementary information). As shown in Fig. S3 (supplementary information), the adsorption isotherms of IB rapidly increased at low IB concentrations (0.0 to 0.1 mg/mL) and quickly reached the equilibrium in all cases. Note that the isotherms were well fitted with the Langmuir equation, in which the maximum adsorption amount for IB was 2.438, 4.405 and 4.875 mg/mg for LRD, HEC and LFN, respectively (Table 1). These clay nanoparticles all showed higher protein-infinity than the other widely used nano-carriers (Table 1)^37,38^.

**Table 1:** Effective IB adsorption amount on four clay nanoparticles, calculated by Langmuir equation.

	LRD	HEC	LFN	AM-41	MCM-41
Maximum absorbance (mg protein/mg NPs)	2.438 ± 0.048	4.405 ± 0.044	4.875 ± 0.107	0.029	0.072

Binding equations for IB and three clay nanoparticles, LRD, HEC and LFN were analysed; equations for BSA and silica nanoparticles, AM-41 and MCM-41 are also listed.

**Figure 2:**
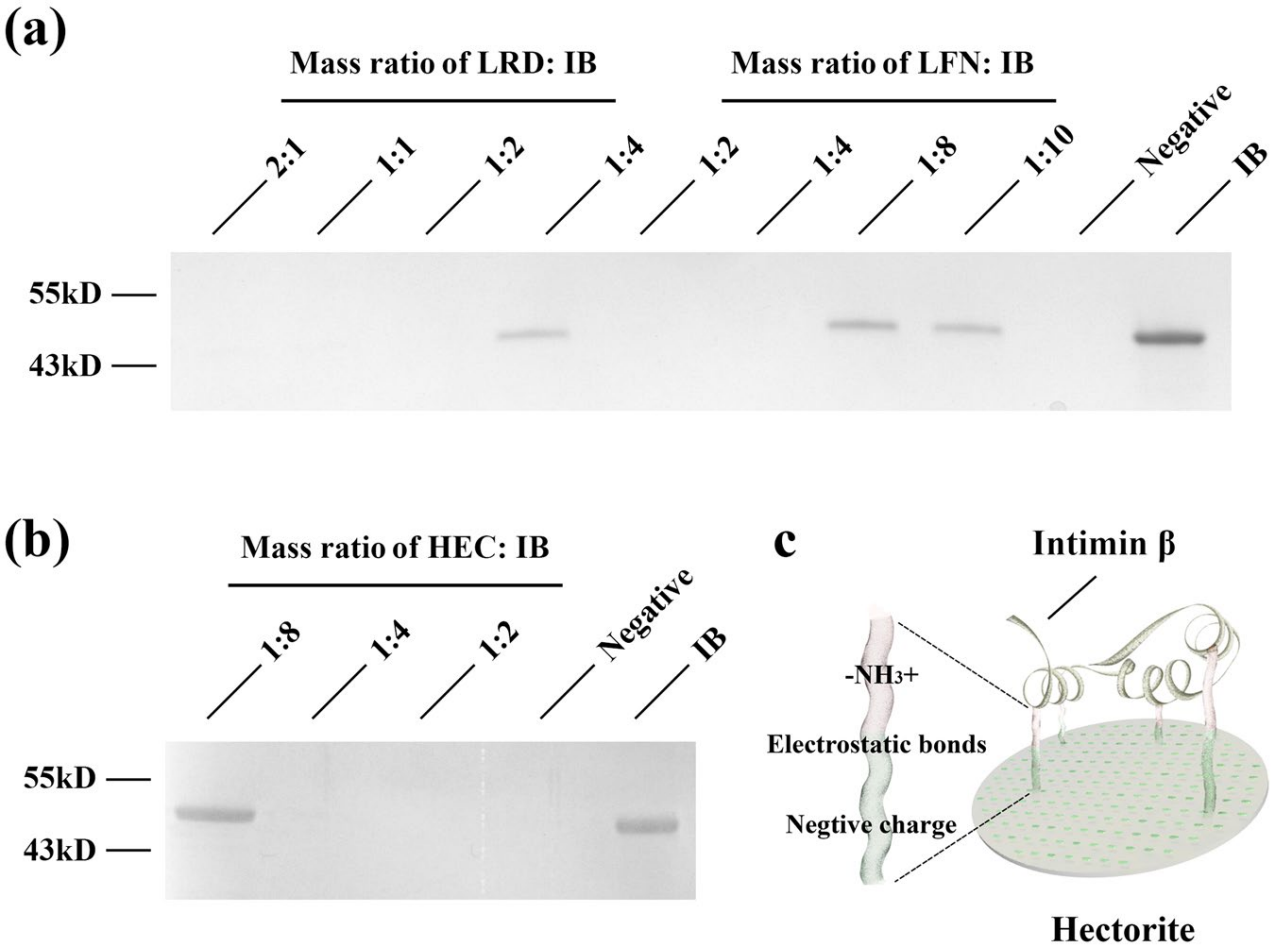
Binding of Intimin β (IB) to clay nanomaterials. SDS-PAGE gels show IB remaining in solution after incubation with (**a**) LRD and LFN, and (**b**) HEC at different mass ratios; the full-length gels were presented in Supplementary Fig. S9; (**c**) Scheme of the interactions between antigen (intimin β; IB) and clay nanoparticle-hectorite, are mediated via electrostatic bonds between –NH_3_+ groups and negative surface charges (hectorite).

Determination of unbound protein in supernatant by SDS-PAGE (Fig. 2a) revealed that LRD was able to immobilize nearly 100% and 70% of IB at the LRD: IB mass ratio of 1:2 and 1:4, respectively. Similarly for LFN and HEC, nearly 100% and 70% of IB was captured at the mass ratio of 1:4 and 1:8 (Fig. 2a,b). These data indicate that these hectorites have high protein loading capacity via efficient affinities for protein-based antigen molecules via electrostatic force (Fig. 2c), consistent to the maximum adsorption amount calculated from the Langmuir equation. More importantly, only 7~9% IB was released from IB-NPs complexes (at mass ratio 4:1) after mixing with 2 × PBS for 2 h, which clearly indicates the high affinity between IB and three hectorites (supplementary Fig. S4).

### Clay nanoparticles with medium size and surface charge induce strong antibody response to IB

**Figure 3:**
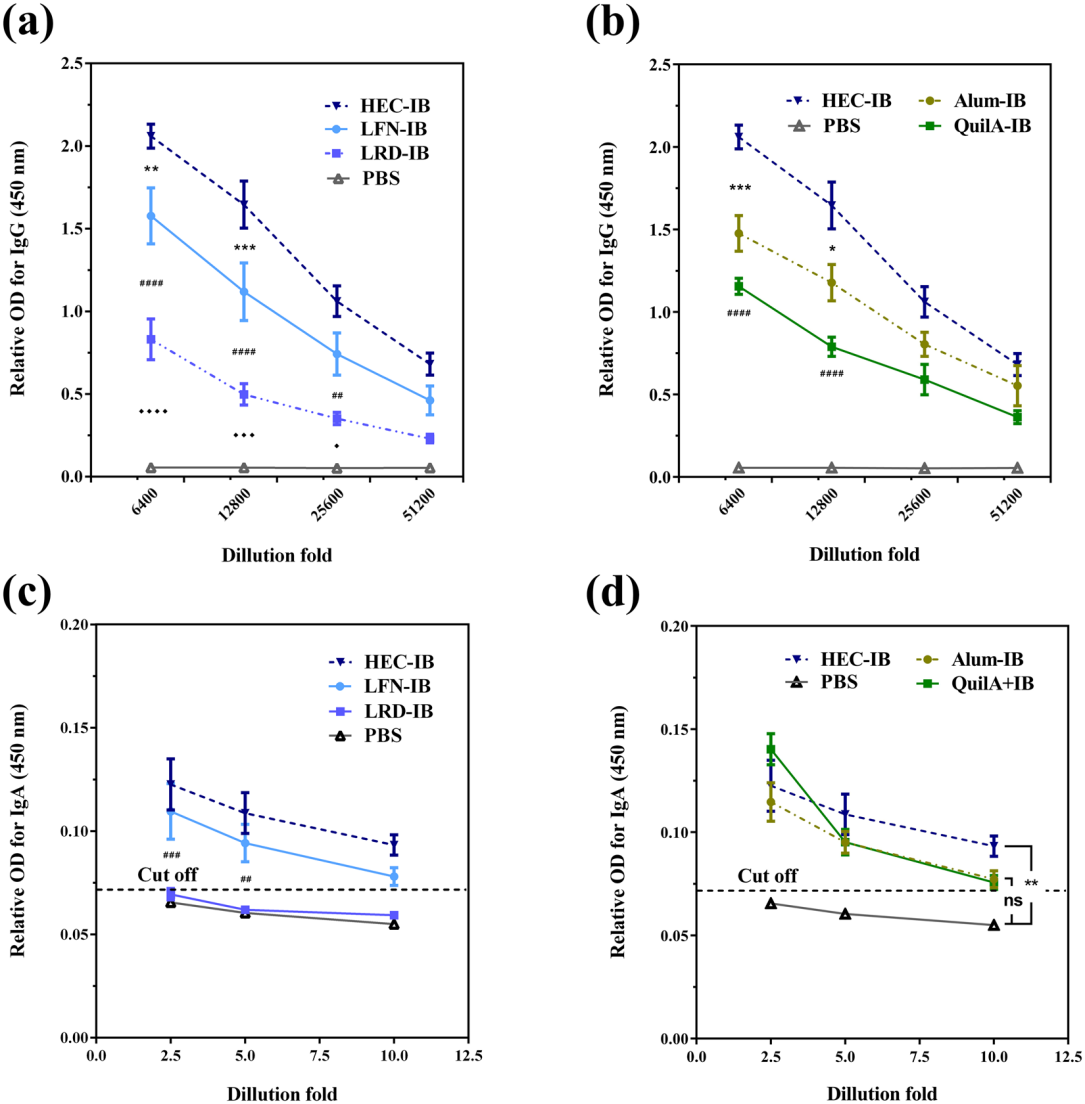
An efficient humoral immune response can be triggered by clay nanoparticle adjuvants. Levels of anti-IB IgG in sera collected from female C57BL/6 J mice (n = 5) 35 days after IB immunisation with (**a**) hectorites LRD, HEC and LFN) or (**b**) best clay nanoparticle-HEC, commercial adjuvants Alum and QuilA, or PBS (negative control) were analysed at serial dilutions from 1:6400 to 1:51200. Specific anti-IB SIgA in faeces at day 49 after IB immunisation (n = 5) with (**c**) three hectorites and (**d**) the most efficient nanoparticle, HEC and commercial adjuvants. Symbols (*/♦/#) in (**a**) and (**c**) represent differences between adjacent two groups, while symbols (_*_/**#**) in (**b**) indicate differences between test nanoparticle and commercial adjuvants (*Alum ^#^QuilA). The cut off was calculated by the formula: Cut-off = mean + 10*SD. Data are expressed as mean ± S.E.M. (n = 5). *P < 0.05; **P < 0.01; ***P < 0.001; and ****P < 0.0001.

To determine the capacity of these nanomaterial/IB complexes as vaccines, mouse immunization was performed. Figure 3a shows that three hectorite-type adjuvants generally promoted production of specific anti-IB IgG. Specifically, the highest IgG level was detected in the group using HEC as adjuvant, and significantly higher than that promoted by LFN and LRD at dilutions of 6400 and 12800 (Fig. 3a). These data suggest that hectorite nanoparticles with the size around 70 nm (e.g. HEC) stimulate the strongest anti-IB antibody response. As also shown in Fig. 3b, the IgG level in mice vaccinated with HEC-IB is significantly higher than that induced by the commercial adjuvants Alum and QuilA at dilutions of 6400 and 12800. In comparison with the optimal LDH nanoaprticles^31^, 73 nm HEC nanoparticles promoted the same level of specific anti-IB IgG (supplementary Fig. S5a).

Secreted anti-IB mucosal IgA (SIgA), the primary antibody responsible for frontline defence against gastrointestinal infections^39^, was examined via ELISA as the key indicator of protective immune responses against EHEC colonization. Figure 3c shows that vaccine adjuvanted with HEC induced the strongest SIgA response, while LFN induced relatively weaker SIgA response and LRD failed to do so. In consistence, the optimal size may be around 70 nm for hectorite nanoparticles as adjuvant in promotion of SIgA secretion. In comparison, mice vaccinated with HEC-IB group seemed to produce the highest level of SIgA (0.093 ± 0.002) at a dilution of 1:10, which was higher than that promoted by commercial adjuvants Alum (0.077 ± 0.005) and QuilA (0.075 ± 0.004), clearly indicating the superior adjuvant activity of HEC (Fig. 3d). The adjuvanticity of 73 nm HEC nanoparticles in provoking SIgA is also comparable to that of the optimal LDH nanoparticles (supplementary Fig. S5b).

In order to ensure the bio-safety of three hectorites in vaccine application, the cytotoxicity assay was conducted. As shown in supplementary Fig. S6, HEC and LFN showed good biocompatibility even at the concentration up to 200 μg/ml. In contrast, small hectorite (LRD) showed some cytotoxicity^40,41^ to cells, with around 70% viability at the concentration of 100 μg/ml. Even thought, three hectorites are much better than some NP adjuvants, such as silica nanovesicle (SV) that caused around 45% cell death at 100 μg/ml^42^. Thus these hectorites are good for animal vaccine application as adjuvants.

### HEC nanoparticle excites effective cellular immune response to IB

**Figure 4:**
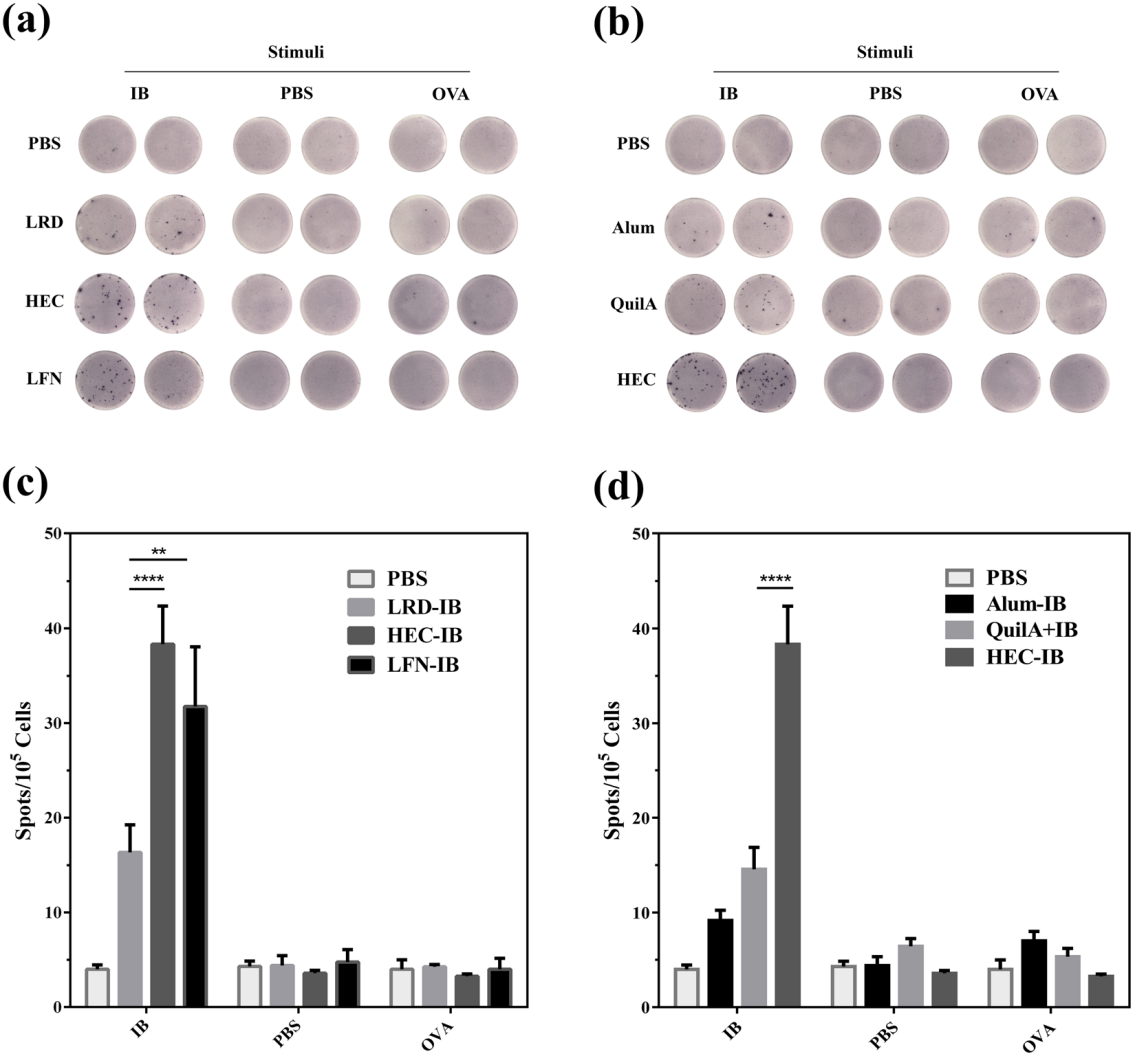
Interferon-γ (IFN-γ) secretion by splenocytes from C57BL/6 J mice (n = 4/group) 49 days after vaccination with IB in combination with commercially available adjuvants (QuilA or Alum), nanoparticles (HEC, LRD or LFN) or PBS alone (control). Splenocytes (1.0 × 10^5^ cells/well) were harvested and co-cultured with IB (specific antigen), OVA (irrelevant antigen) or PBS (no antigen) for 40 h, then secretion of interferon-γ (IFN-γ) determined by ELISPOT assay. Photomicrographs show IFN-γ expressing splenocytes in response to (**a**) three hectorites and (**b**) best clay nanoparticle or commercial adjuvants; bar charts (**c**) and (**d**) illustrate the number of IFN-γ secreting spots in each vaccination group after stimulation with antigen (IB or OVA) or PBS. Data are expressed as mean ± S.E.M. (n = 4). *P < 0.05; **P < 0.01; ***P < 0.001; and ****P < 0.0001.

The capabilities of three hectorite nanoparticles to promote the cellular immune response to IB were determined by ELISPOT assay. As shown in Fig. 4a and c, the number of interferon-γ (IFN-γ) secreting splenocytes was highest for mice receiving HEC as adjuvant (38.3 ± 3.9 spots per 10^5^ cells), followed by LFN (31.8 ± 6.2 spots per 10^5^ cells) and LRD (16.3 ± 2.9 spots per 10^5^ cells) (Fig. 4c). These data have also shown that the medially sized hectorite nanoparticles (HEC) promote the highest cellular immune response.

As a Th2-biased adjuvant^25^, Alum seemed not to induce a valid IFN-γ response (9.1 ± 0.9 per 10^5^ cells) to antigen IB. The commonly used adjuvant QuilA only induced a weak IFN-γ response (14.2 ± 2.7 spots per 10^5^ cells), much weaker than that induced by the best sized hectorite (HEC) (Fig. 4b and d). This further indicates the capability of HEC as efficient Th1 adjuvant. As expected, neither the control protein (OVA) nor PBS (no-antigen control) induced effective IFN-γ secretion (Fig. 4).

### Clay nanoparticle with the optimized size promotes protection against EHEC O26 attachment to ruminant cells

**Figure 5:**
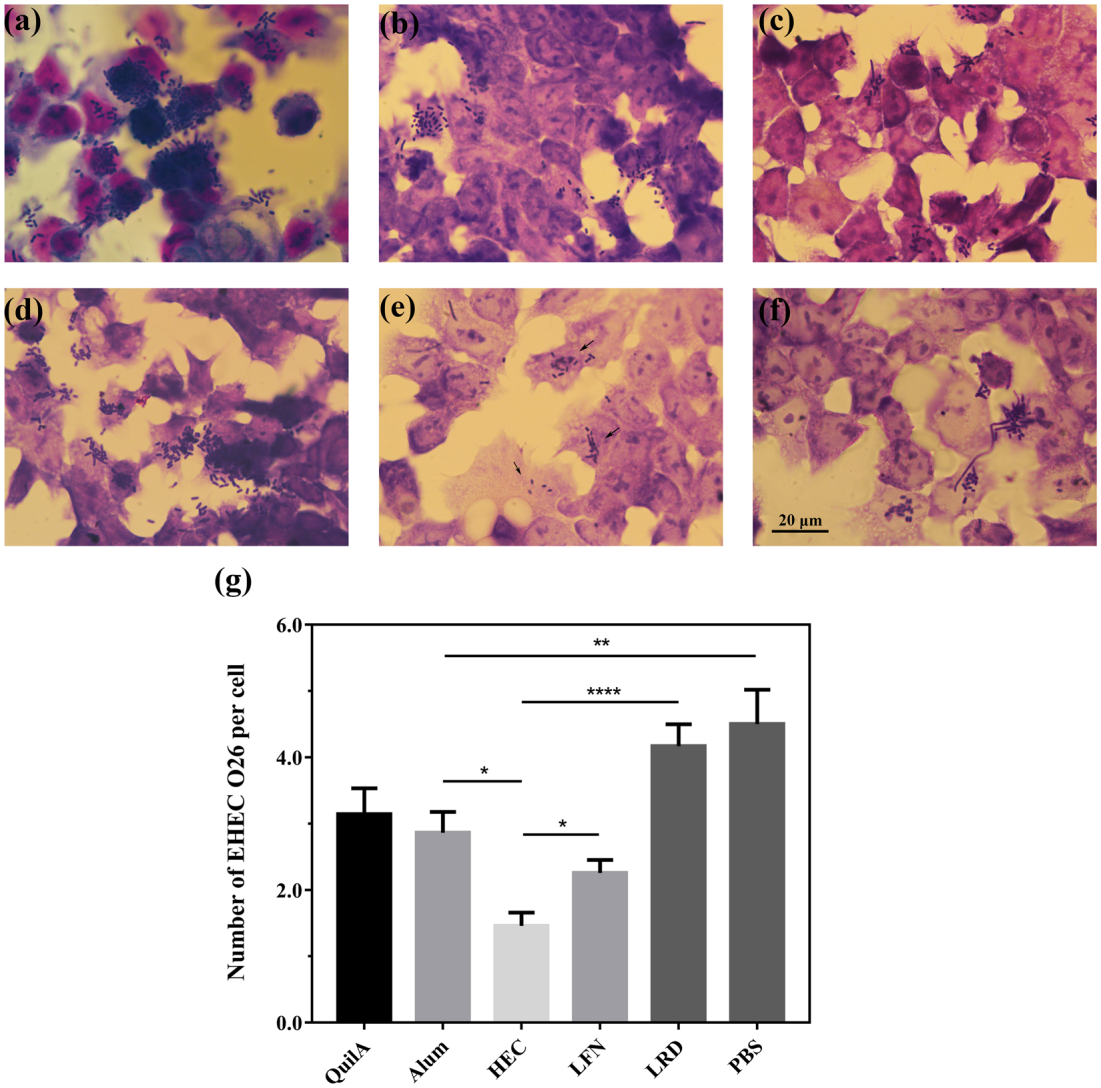
Nanoparticle adjuvants promote an efficient SIgA response to IB vaccination, capable of inhibiting EHEC O26 attachment to ruminant cells. MDBK cells were incubated with EHEC O26 cells in the presence of faecal SIgA extracts from mice vaccinated with IB in the presence of different adjuvants: (**a**) PBS, (**b**) QuilA, (**c**) Alum, (**d**) LRD, (**e**) HEC and (**f**) LFN. Cells were then stained with GIEMSA, and (**e**) the number of *Escherichia coli* attached to each cell calculated by morphometric technique. Data are expressed as mean ± S.E.M. (n = 4/group). *P < 0.05; **P < 0.01; ***P < 0.001; and ****P < 0.0001. Scale bar = 20 µm.

Diarrheagenic *Escherichia coli* including EHEC infection of bovine cells is majorly mediated by intimins^14^. To further determine vaccine efficacy mediated by clay nanoparticles, SIgA secreted in the faecal extract was tested for the ability to prohibit EHEC O26 attachment to Madin-Darby bovine kidney (MDBK) cells. As shown in Fig. 5, the SIgA extract from mice receiving HEC-adjuvanted vaccine offered much better protection of host cells against EHEC O26’s infection. For example, only 1.4 ± 0.2 adherent bacteria/cell was observed for SIgA extract from HEC group, significantly lower than that induced by LRD (4.1 ± 0.3 adherent bacteria/cell), LFN (2.2 ± 0.2 adherent bacteria/cell), and two commercial adjuvants (3.1 ± 0.4 and 2.8 ± 0.3 adherent bacteria/cell for QuilA and Alum group of mice, respectively). Few MDBK cells escaped EHEC O26 infection following incubation with the extract from the PBS control group (4.5 ± 0.5 adherent bacteria/cell, Fig. 5a and g), further supporting the specificity of SIgA extracted in the faecal via this binding assay. These observations are consistent with SIgA ELISA results (Fig. 3c and d), and thus indicate that HEC nanoparticles induce much better protection against EHEC O26’s infection than commercial adjuvants.

### Optimally-sized clay nanoparticle also promotes protection against EHEC O26 infection of human intestinal cells

**Figure 6:**
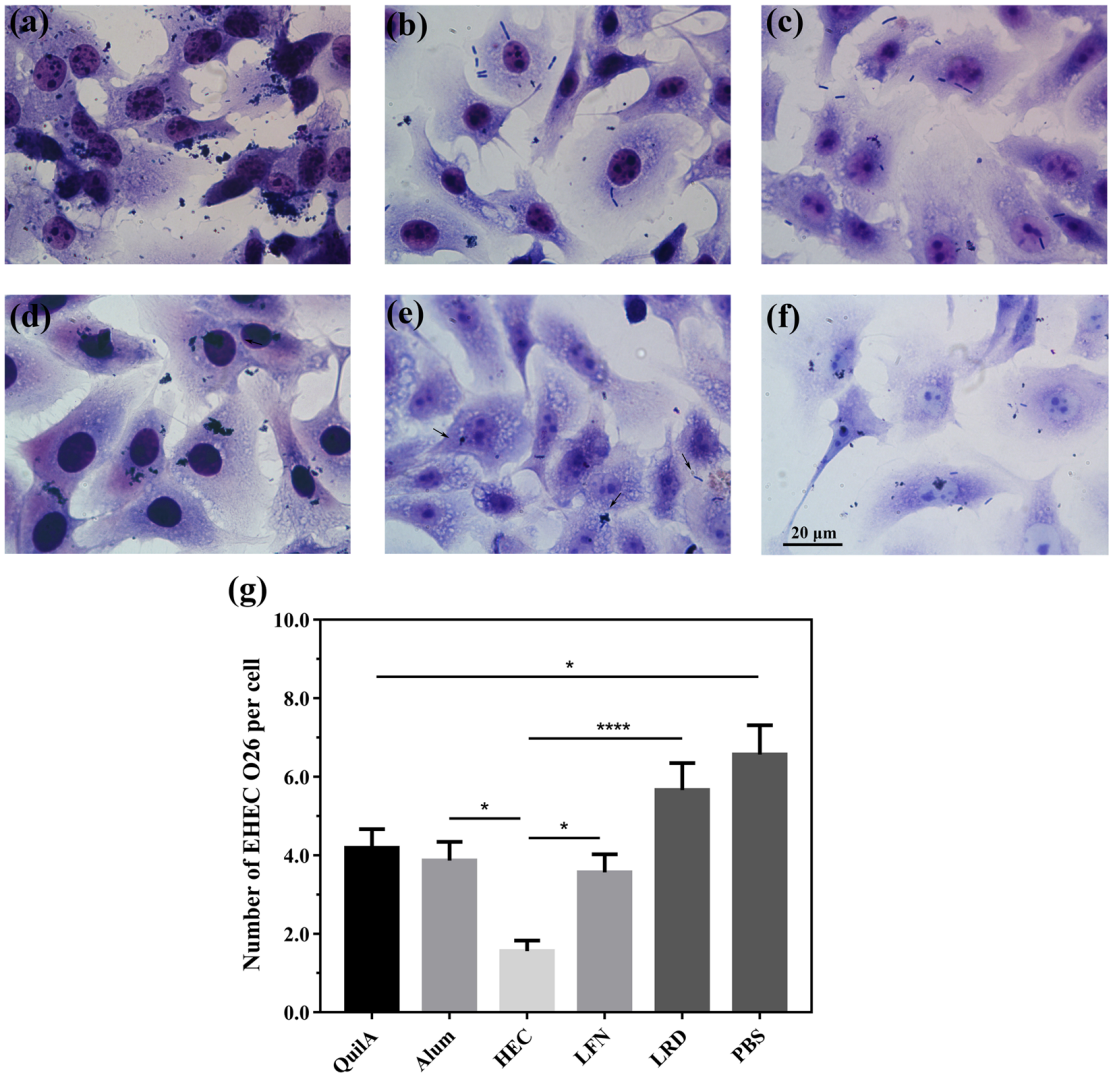
SIgA extracts from mice vaccinated with IB in combination with clay nanoparticle adjuvant effectively block EHEC O26 attachment to human intestinal cells. HRT-18 cells were infected with EHEC O26 cells in the presence of faecal-SIgA extracts from different vaccination groups. Photomicrographs (a–d) show GIEMSA staining of cells treated with fecal-SIgA extracts from mice vaccinated with IB plus (**a**) PBS, (**b**) QuilA, **(c**) Alum, (**d**) LRD, (**b**) HEC and (**f**) LFN; Histograms (**e**) show the number of bacteria attaching to each cell calculated by morphometric technique. Data are expressed as mean ± S.E.M. (n = 4). *P < 0.05; **P < 0.01; ***P < 0.001; and ****P < 0.0001. Scale bar = 20 µm.

SIgA in the faecal extract induced by different vaccine formulations was also tested for its ability to prohibit intimin-mediated EHEC O26 infection of the human colorectal adenocarcinoma cell line, HRT-18. Similar to MDBK cells, HRT-18 cells incubated with SIgA secretions from HEC vaccination groups showed a much more reduced EHEC O26 infection (1.5 ± 0.3 adherent bacteria/cell; Fig. 6) in comparison with that from QuilA (4.1 ± 0.5 adherent bacteria/cell) and Alum (3.8 ± 0.5 adherent bacteria/cell) groups. SIgA from the LFN vaccination group (3.5 ± 0.5 adherent bacteria/cell) mediated better protection than that from the LRD group (5.6 ± 0.7 adherent bacteria/cell; Fig. 6). These data indicate that (1) 70-nm hectorite nanoparticles induce the maximum protection and (2) HEC is efficient adjuvant and able to induce effective protection for ruminant and human cell against EHEC O26 invasion. The protection capability of HEC-adjuvanted vaccine is noted to be comparable to that of optimized LDH nanoparticles (Supplementary Fig. S7), further indicating the significant adjuvanticity of HEC and LDH nanoparticles and the particle size effect.

### Clay nanoparticles effectively excite macrophages to express co-stimulatory cytokines

**Figure 7:**
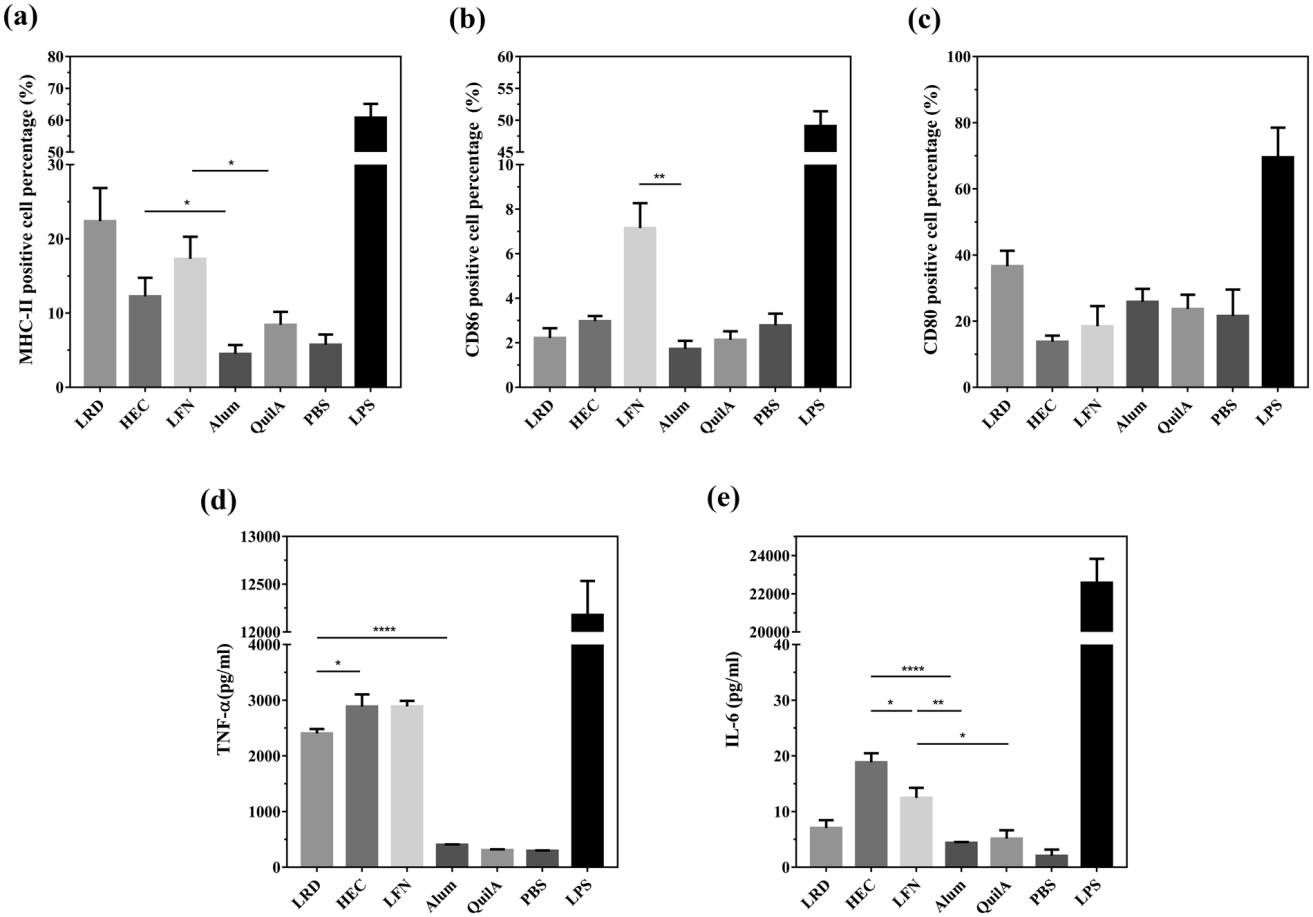
Clay nanoparticles are able to induce macrophage maturation and cytokine secretion in response to antigen stimulation. RAW 264.7 macrophages were stimulated just with clay nanoparticles (with final concentration at 10 μg/mL), commercial adjuvants (final concentration of Alum and QuilA at 10 μg/mL and 0.5 μg/mL respectively), PBS and LPS (with final concentration at 100 ng/mL). Then the surface expression of (**a**) MHC-II, (**b**) CD86 and (**c**) CD80 were examined by flow cytometry, while the secreted cytokines (**d**) TNF-α and (**e**) IL-6 in culture supernatants were assayed via ELISA. Data are expressed as mean ± S.E.M. (n = 4). *P < 0.05; **P < 0.01; ***P < 0.001; and ****P < 0.0001.

The effect of pure clay nanoparticles on antigen presenting cell (APC) maturation was further examined in RAW 264.7 macrophage line. As shown in Fig. 7a, three nanoparticles were able to induce expression of MHC-II at the moderate level (from 12.3% to 22.4% positive cells), which is higher than that induced by the commercial adjuvant Alum (4.5% positive cells) and QuilA (8.4% positive cells). Similarly, more cells expressed CD86 (co-stimulator B7-2) after incubation with pure clay nanoparticles compared with various control groups (Fig. 7b). There were no significant differences in expression of CD80 (B7-1) among all adjuvants (Fig. 7c).

As shown in Fig. 7d, three hectorites dramatically stimulated secretion of IFN-γ at 2000 to 3000 pg/mL when 10 μg/mL of nanoparticles was included in incubation medium. However, Alum and QuilA induced limited responses (IFN-γ < 400 pg/mL) under the same conditions. At the same concentration of nanoparticles, IL-6 levels were higher after incubation with HEC (18.9 pg/mL) and LFN (12.5 pg/mL) than that with LRD (7.1 pg/mL), QuilA (5.1 pg/mL) and Alum (4.4 pg/mL) (Fig. 7e).

Interestingly, incubation of cells with adjuvants in the presence of antigen (IB) did not obviously change the expression profiles of co-stimulatory and cytokines (Supplementary Fig. S8), indicating that their expressions are primarily triggered by these clay nanoparticles.

## Discussion

In the present work, we reported cationic clay nanoparticles as effective vaccine nano-adjuvants in exciting immune responses and protection against EHEC challenge. Hectorites with three different sizes were applied in vaccine formulations against IB, while HEC immunized mice with the most comprehensive immune barrier (by producing higher level of IgG, IFN-γ and SIgA), preventing EHEC infection. In combination with our previous study^31^, these findings suggest that having the medium diameter and surface charge, HEC (with medium diameter of around 73~77 nm and zeta potential of −33.5~−41.1 mV) is able to achieve safe, durable and high immune response superior to the commercial adjuvants, which could be attributed to peculiar properties of the clay-IB nanocomplexes.

The first property is the high binding capacity of clay nanoparticles for IB. Three hectorites are able to bind 2.43~4.87 mg (IB)/mg (hectorite) (Table 1), which is significantly higher than that of other reported nanoparticles (e.g. chitosan and mesoporous silica nanoparticles)^27,38,43^. This high antigen loading can be due to strong electrostatic interaction between proteins and clay nanoparticle as well as the large surface area (Fig. 2c). In comparison, hydrophilic IB may interact with Alum or QuilA via the relatively weak bonds, such as H-bonds and van der Waal bonds, and the binding capacity of these commercial adjuvants is relatively low. In comparison, HEC nanoparticles had a moderate zeta potential and thus a moderate binding capacity (Table 1), which may benefit the promotion of immune responses.

The second property is facilitation of antigen cellular uptake via clay nanoparticles. As reported in our previous paper, HEC nanoparticles delivered much more albumin to macrophage cells^31^. The IB uptake by antigen presenting cells (APCs) in mice is thus facilitated similarly by clay nanoparticles. The sizes of hectorites are all below 200 nm even after binding with IB (data no shown), which is suitable for internalization by dendritic cells (DCs), the representative of APCs^44^. Moreover, this size range is similar to the viral size, which might allow clay-antigen nano-complexes to build danger signal and further provoke immune responses^45^. Relatively, the medium size of HEC nanoparticles may more efficiently facilitate the uptake of antigen and presentation.

Another crucial property is their stimulation capability for APC maturation in comparison with the commercial adjuvant QuilA or Alum. Previously, LDH, one type of clay nanoparticles has been shown to stimulate macrophage responses^46^, which would dramatically contribute to their remarkable adjuvanticity (supplementary Figs S5 and S7). Thus, our current studies investigate the capability of hecorites in stimulating APCs, and demonstrate that three hectorites excited higher expression of MHC-II, CD86 and TNF-α in macrophage cell. In particular, HEC nanoparticles stimulated abundant secretions of TNF-α and IL-6, i.e. HEC induced both potent Th1 and Th2 immune responses. Relatively, LRD and LFN stimulated macrophage cells to secret a limited amount of IL-6, i.e. they induced a biased Th1 immune response. Thus our investigation clearly indicates that clay nanoparticles stimulate APC maturation, which is expected to take place in mice after vaccination with clay-IB nanocomplexes.

As often suggested, the so-called depot-effect^47^ is largely responsible for the sustained stimulation and the prolonged immune responses^31^ after s.c. injection. In the current study, s.c. injection of clay-IB nanocomplexes likely leads to accumulation of clay-IB aggregates at the injection site, resulting in sustained immune responses via continuous internalization by APCs, where the primary medium clay particle size may bring more benefits^44^. Such parenteral vaccine (s.c. vaccination) would primarily induce secretion of serum antibodies, but the anti-bacterial protection to two cell lines provided by clay nanoparticle vaccine groups might be provoked by activated APCs that migrate to draining lymph nodes, intestinal and neighbouring lymphoid tissues such as mesenteric lymph nodes for producing secretory immunoglobulin (including SIgA)^39,48–50^, which has also been observed previously^51^. Moreover, HEC might increase the expression of pIgR/SC via relative higher interferon-γ (IFN-γ) production compared with commercial adjuvants^52^, and promote the SIgA transportation from mesenteric lymph nodes into the gut. In brief, the optimally sized HEC nanoparticles effectively stimulate APCs and promote comprehensive immune responses against pathogenic *E. coli* invasion.

In conclusion, this study demonstrates the high potential of clay nanoparticles as effective nano-adjuvants to enhance immune responses to next generation vaccine antigens. Overall, clay nanoparticles (HEC) with the medium size and zeta potential demonstrate superior adjuvant activity compared with commonly used adjuvants QuilA and Alum, inducing both strong cellular and humoral immune responses to the antigen. Our results promise an efficient, safe and executable vaccine approach in controlling EHEC O26 infection.

## Methods

### Preparation and characterization of nanoparticles

Synthetic hectorites, Laponite WXFP (HEC;[(Si_8_Mg_5.34_Li_0.66_)O_20_(OH)_4_]·Na_0.66_),laponite RD(LRD;[(Si_8_Mg_5.55_Li_0.43_)O_18.45_(OH)_7.36_]·Na_0.73_) and Laponite FN (LFN; [(Si_8_Mg_4.17_Li_1.27_)F_1.7_ O_18.01_(OH)_5.98_]·Na_0.54_), were supplied by Rockwood Additives Inc., USA. After a two-day calcination at 200 °C for sterilization, samples were dissolved in endotoxin-free water, with gentle ultrasonication.

The particle size distributions and zeta potential of these nanoparticles in aqueous suspensions were measured by photon correlation spectroscopy (PCS, Nanosizer Nano ZS, MALVERN Instruments). Nanoparticle suspensions were mounted on copper grid, air dried and TEM images obtained with JEOL 1010 (with acceleration voltage 100 kV) and JSM-2100 (acceleration voltage of 200 kV) transmission electron microscopes (TEM). Composition and crystal structures of clay nanoparticles were characterized with X-ray diffraction (XRD) patterns (Rigaku Miniflex X-ray diffractometer) and Fourier transformed infrared (FTIR) spectra (Nicolet 6700 FTIR spectrometer).

### IB production and adsorption onto clay nanoparticles

Recombinant intimin β (IB), C-terminal fragment, was purified as described previously^31^. To obtain adsorption isotherms for IB with these nanoparticles, suspensions were added (at mass ratios of 1:1 to 1:10) into IB solution with continuous stirring. Unbound IB was separated by centrifugation at 21000 g for 20 min, and the concentration in collected supernatants measured by NanoDrop (Thermo Scientific) (λ = 280 nm). The isotherms of IB binding to clay nanoparticles were simulated by fitting to the classic Langmuir model using statistical software (GraphPad Prism 7). To determine the maximal binding mass ratio of LRD/LFN to IB (100% adsorption of IB to nanoparticles), free IB in supernatants was also analyzed by SDS-PAGE.

### Animal model

Eight weeks old female C57BL/6 J mice were purchased from the University of Queensland Biological Resources, and housed under specific pathogen-free conditions. All procedures were approved by the University of Queensland Animal Ethics Committee Guidelines and conformed to the Australian Code of Practice for the Care and Use of Animals for Scientific Purposes (8th edition, 2013).

### Animal vaccination

Mice were grouped (5 mice per group) randomly. Two subcutaneous injections (60 μL per injection) including one primary injection and one booster injection 3 weeks later were administered to the nuchal region. Vaccination groups were as follows: Group 1, PBS (control group); Group 2, Alum (Aluminium hydroxide gel, InvivoGen)-IB (160 μg:5 μg); Group 3, QuilA-IB (5 μg:5 μg); Group 4, HEC-IB (160 μg:5 μg); Group 5, LFN-IB (160 μg:5 μg) and Group 6, LRD-IB (160 μg:5 μg). Group 7, LDH-IB (160 μg: 5 μg). Mice were bled from the tail tip at day 35 after primary vaccination, then euthanised by CO_2_ inhalation at day 49, blood obtained by cardiac puncture, and spleens and faeces harvested. Blood samples were allowed to clot, then centrifuged (4 °C) for 20 min at 1500 g to recover the serum, which was then stored at −80 °C until required.

### Extraction of intestinal mucosal antibodies

To obtain intestinal mucosal SIgA, fresh faeces were recovered from excised intestine at day 49 and weighed carefully. Pre-cooled PBS was added to each sample to a concentration of 0.1 g/mL, then samples centrifuged at 16000 *g* (4 °C) for 20 min. Supernatants containing mucosal SIgA were stored at −80 °C for ELISA and test for EHEC colonization on two cell lines.

### Detection of IB-specific antibody responses

The levels of serum IgG and secretory (S)IgA in faecal extracts were determined by ELISA. In brief, 96 well plates (Nunc, Maxisorb, Roskilde, Denmark) were coated with IB by adding 100 µl of IB solution (1 μg/mL in sodium carbonate buffer (0.1 M; pH 9.5), and incubating overnight at 4 °C. Wells were washed with PBS containing 0.1% Tween-20 (PBS-T), and then blocked by incubation with PBS-T containing 5% bovine serum albumin (Sigma-Aldrich) for 1 h at room temperature with shaking gently. Wells were washed with PBS, followed by adding of 100 μL of serum (diluted from 1:6400 to 1:51200 in PBS) or faecal extract (diluted from 1:2.5 to 1:10 in PBS) and incubating for 1 h at room temperature. After further washing, goat anti-mouse IgG-HRP or IgA-HRP conjugates (diluted to 1:2000 in PBS) were added into wells for 1 h. Wells were washed and developed by incubating with TMB substrate (100 μL/well, Thermo Scientific) for 15 min at room temperature, followed by adding H_2_SO_4_ (2 N, 50 μL) to stop the chromogenic reaction. The optical density at 450 nm was measured on a SpectraMax® M5 microplate reader.

### ELISPOT assay for interferon-γ (IFN- γ) secretion by splenocytes

Spleens were aseptically harvested at necropsy at day 49, and placed in ice-cold RPMI-1640 (Gibco) medium containing 100 mg/mL streptomycin, 100 U/mL penicillin G, 2 mM L-glutamine and 10% fetal bovine serum (heat inactivated, FCS). Single cell suspensions were prepared by gently pushing through 70 μm nylon cell strainers (BD Bioscience). Cells were pelleted (400 g, 5 min, 4 °C), then resuspended in red cell lysis buffer (5 mL, 0.15 M NH_4_Cl, 10 mM KHCO_3_ and 0.1 mM Na_2_-EDTA) for 10 min on ice. After two washes with RPMI, splenocytes from each mouse were seeded (1.0 × 10^5^ cells/well) into duplicate wells of polyvinylidene fluoride (PVDF) pre-coated ELISPOT plates (containing monoclonal interferon-γ capture antibody; Mabtech). The cells were cultured in complete RPMI-1640 at 37 °C in 5% CO_2_ for 40 h in the presence of specific antigen (IB; 5 μg/mL in PBS), non-specific antigen ovalbumin (OVA, Grade V; 5 μg/mL in PBS), or PBS (negative control). ELISPOT assays were performed according to the manufacturer’s instructions, and interferon-γ-producing splenocytes detected using an AID ELISPOT reader system.

### Inhibition of enterohaemorrhagic escherichia coli attachment to target cells

Madin-Darby bovine kidney (MDBK; ATCC® CCL-22™) and human colorectal adenocarcinoma (HRT-18; ATCC® CCL-244™) cell lines were cultured in Dulbecco’s Modified Eagle’s Medium (DMEM) and RPMI 1640 medium (respectively) with 10% FCS, 100 mg/mL streptomycin and 100 units/mL penicillin (Gibco® life technologies™). Enterohaemorrhagic *Escherichia coli*, EHEC O26 strain (N39) was aseptically spread on LB-agar plates (Amresco, Solon, USA) without antibiotics, and cultured overnight at 37 °C. A single clone was picked, inoculated in LB Miller broth (5 mL), grown in static culture overnight, and then harvested for assays.

MDBK and HRT-18 cells were seeded (at 2 × 10^5^ cells/well) into 12 well plates containing sterile cover slips, and cultured in complete medium overnight at 37 °C in 5% CO_2_. Plates were washed with warm PBS three times and then added with SIgA-EHEC O26 mixtures. The SIgA-EHEC O26 mixture were faecal extracts diluted 1:10 in culture medium (600 μL, DMEM or RPMI, without FCS or antibiotic) and mixted with 1 μL (for MDBK) or 20 μL (for HRT-18) of EHEC O26 culture at OD = 0.25. These mixtures were incubated for 30 min at 37 °C, with three-time mixing for well dispersion. After that, the mixtures were added and incubated with the cells for 3 h. Supernatants were removed and cells washed three times with warm PBS. Then culture medium (RPMI or DMEM) containing SIgA (1/10 dilution) and 10% FCS was added to plate for a further 3-h incubation. Plates were washed five times with warm PBS, then coverslips stained with GIEMSA and the number of E coli attached/cell counted in five random fields for each coverslip.

### Analysis of macrophage cytokine production

The mouse macrophage cell line (RAW 264.7; ATCC® TIB-71™) was seeded in 12 well plates at a density of 3 × 10^5^ cells/well, and grown to approximately 60% confluence. Plates were washed and supplied with fresh culture medium containing clay nanoparticles at a concentration of 10 μg/mL. Control wells received Alum (10 μg/mL), QuilA (0.5 μg/mL), LPS (100 ng/mL) or PBS. In order to determine the effect of nanoparticles as vaccine adjuvants, nanoparticle-IB complexes (mass ratio 32:1), Alum-IB (mass ratio 32:1), QuilA-IB (1:1) or IB alone were added to the culture media to assess the effect of IB at a concentration 0.5 μg/mL in all cases. After 24 h incubation, cells were harvested for flow cytometry analysis, and the medium recovered and centrifuged at 20000 g for 20 min at 4 °C. Supernatants were stored at −80 °C for later measurement of secreted co-stimulatory cytokines by ELISA according to the manufacturer’s instructions (Mouse TNF-α/IL-6 ELISA development kit, MABTECH).

### Flow cytometry analysis for membrane proteins MHC II, CD86 and CD80

Macrophages were washed with PBA solution (PBS with 0.2% bovine serum albumin, 0.1% sodium azide, pH: 7.4) and Fc receptor binding blocked by incubation with 2% TruStain fcX™ (anti-mouse CD16/32; Biolegend, San Diego, CA) in PBA for 15 min on ice. Cells were then stained with Abs to mouse CD80, CD86 and I-Ek (MHC-II) (BioLegend) for 30 min on ice. After washing, cells were fixed in 2% PFA and analyzed on an Accuri C6 flow cytometer (BD Biosciences, Franklin Lakes, NJ).

### Statistical analysis

Data were analyzed by one-way or two-way ANOVA with Bonferroni post-tests using GraphPad Prism 6.0 software; a P value < 0.05 was considered statistically significant. *P < 0.05; **P < 0.01; ***P < 0.001; and ****P < 0.0001.

## Electronic supplementary material

Supplementary Information
