# Supplementary material for: Efficient induction of comprehensive immune responses to control pathogenic E. coli by clay nano-adjuvant with the moderate size and surface charge
Source: Sci Rep. 2017 Oct 17;7:13367. doi: 10.1038/s41598-017-13570-2 (PMC5645426; doi:10.1038/s41598-017-13570-2)
Supplement: Supplementary file 1 — Supplementary Information [file 41598_2017_13570_MOESM1_ESM.pdf]

Efficient induction of comprehensive immune responses to control pathogenic E.coli by clay nano-adjuvant with the moderate size and surface charge

Weiyu Chen[1], Huali Zuo[1], Timothy J. Mahony[2], Bing Zhang[3], Barbara Rolfe[1], and Zhi Ping Xu[1]**

[1]Australian Institute for Bioengineering and Nanotechnology, University of Queensland, St Lucia, QLD 4072, Australia (Email: gordonxu@uq.edu.au). [2]Queensland Alliance for Agriculture and Food Innovation, The University of Queensland, St Lucia, QLD 4072, Australia (Email: t.mahony@uq.edu.au). [3]Department of Agriculture and Fisheries, Brisbane City, QLD 4000, Australia.

Supplementary InformationTable S1. Zeta potential of clay nanoparticles in water. Mass ratio of NP: IB was 32:1 except of QuilA+IB which was applied 1:1 mass ratio of NP: IB.

	**HEC**	**LRD**	**LFN**	**Alum**	**QuilA**
			**In water (mV)**		
NP	-33.5 ± 1.2	-19.0 ± 3.1	-55.3 ± 2.1	17.9 ± 0.4	-2.5 ± 0.6
NP+IB	-25.1 ± 1.4	-15.8 ± 2.4	-29.6 ± 1.1	16.9 ± 0.4	2.8 ± 0.4

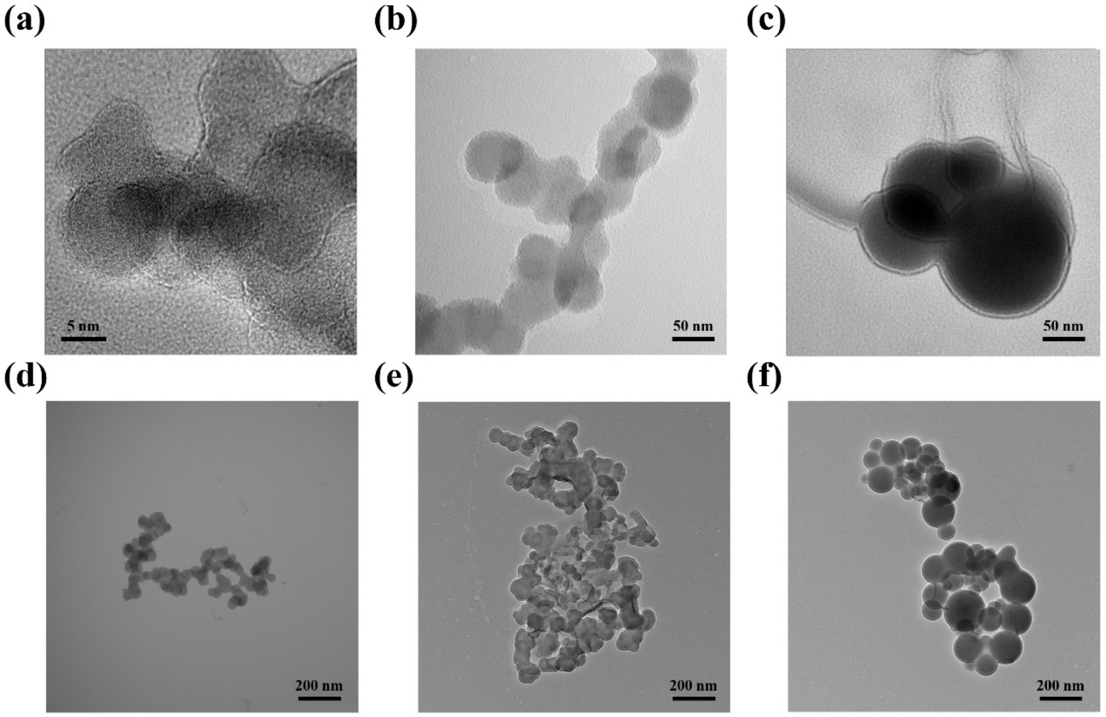

Supplementary Information Figure S1. TEM image of (a, d) LRD, (b, e) HEC and (c, f) LFN.

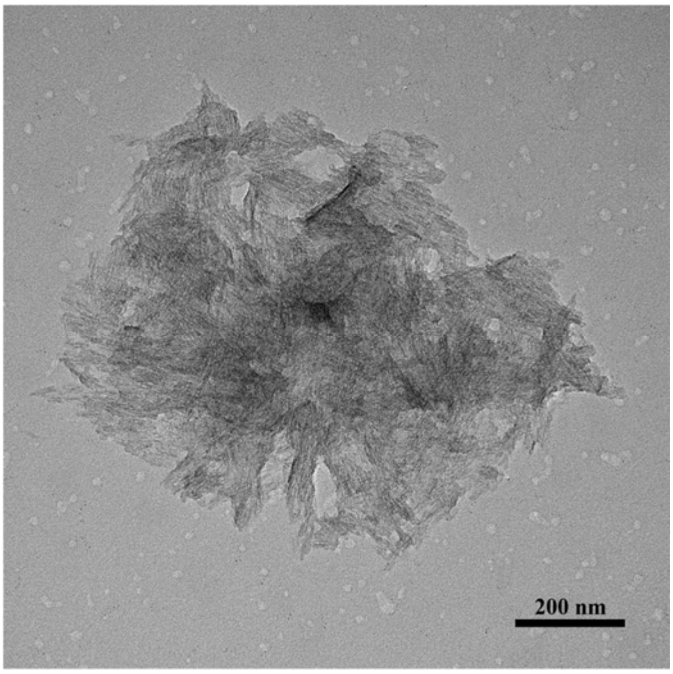

Supplementary Information Figure S2. TEM image of particle in alum hydroxide gel, scale bar = 200 nm.

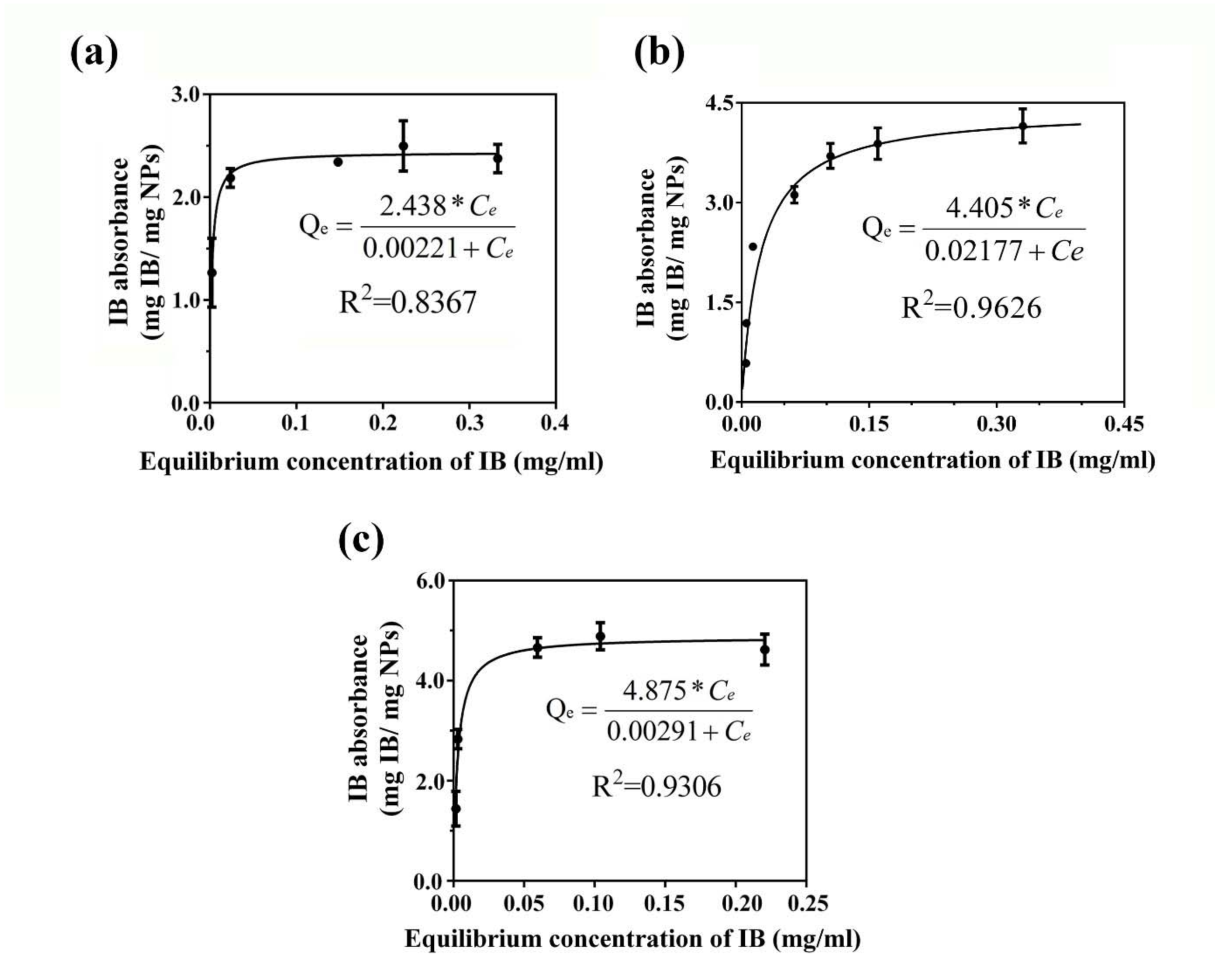

Supplementary Information Figure S3. Langmuir adsorption isotherms of IB on (**a**) LRD, (**b**) HEC and (**c**) LFN nanoparticles. Data are expressed as mean ± S.E.M. (n=4).

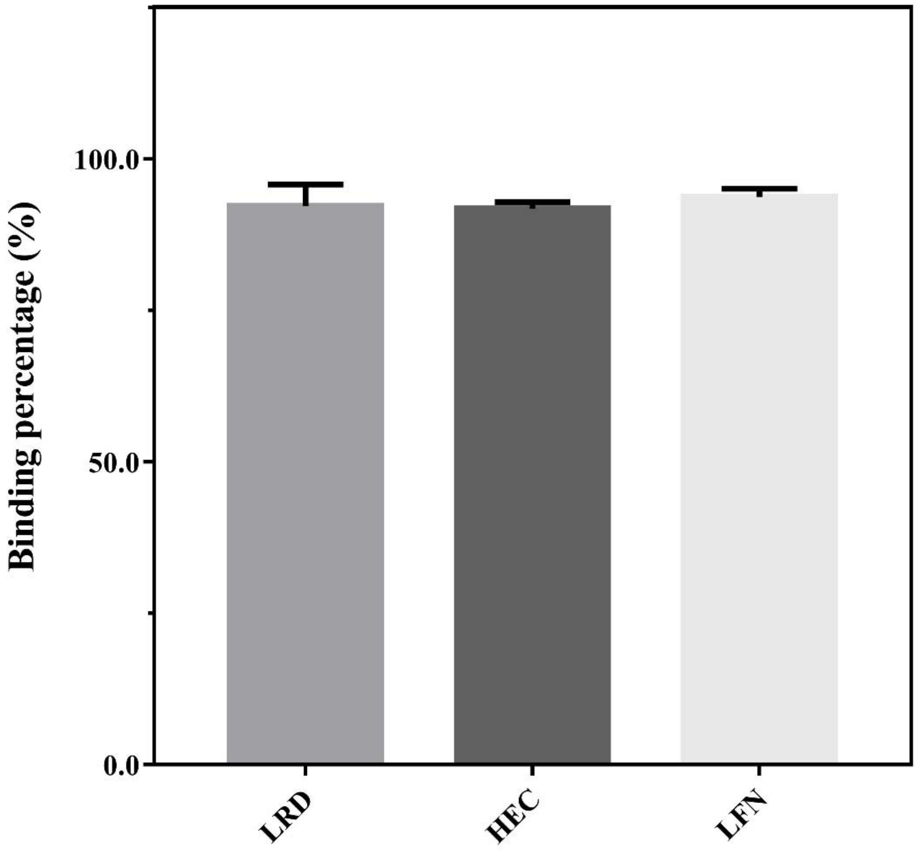

Supplementary Information Figure S4. Binding assays of three sized hectorites. Three hectorites were mixed with IB first, then mixed with 2xPBS for 2 h. The binding efficiency was evaluated via detection of free IB in supernatant. Data are expressed as mean ± S.D. (n=5).

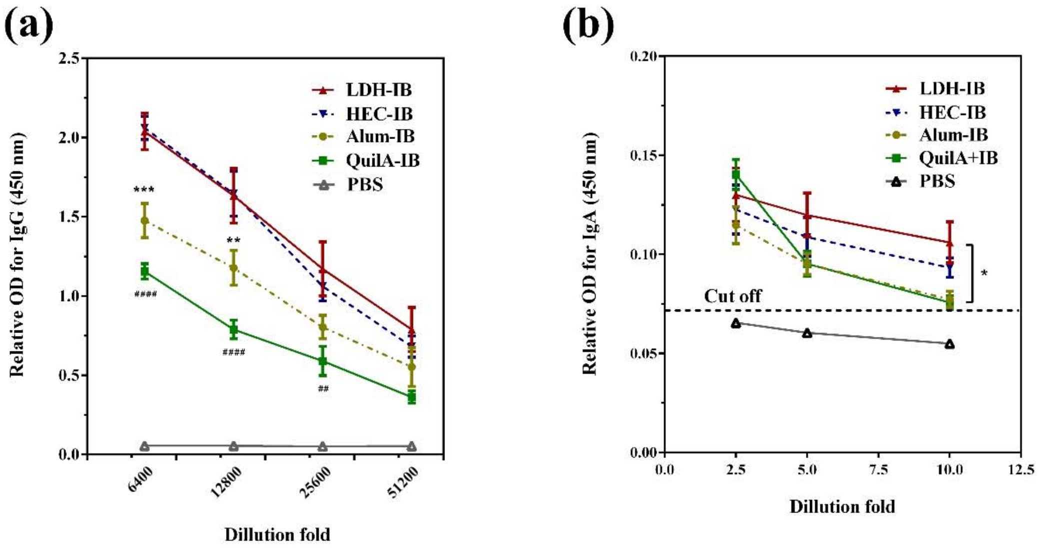

Supplementary Information Figure S5. **Specific anti-IB IgG and SIgA** Levels of anti-IB IgG in sera collected from female C57BL/6J mice (n=5) 35 days after IB immunisation with (**a**) LDH, HEC and commercial adjuvants Alum and QuilA, or PBS (negative control) were analysed at serial dilutions from 1:6400 to 1:51200. Specific anti-IB SIgA in faeces at day 49 after IB immunisation (n=5) with (**b**) LDH, HEC and commercial adjuvants. Symbols (*/#) in a) indicate differences between test nanoparticles and commercial adjuvants (*: Alum #: QuilA). The cut off was calculated by the formula: Cut-off = mean + 10*SD. Data are expressed as mean ± S.E.M. (n=5). *, P < 0.05; **, P < 0.01; ***, P < 0.001; and ****, P<0.0001.

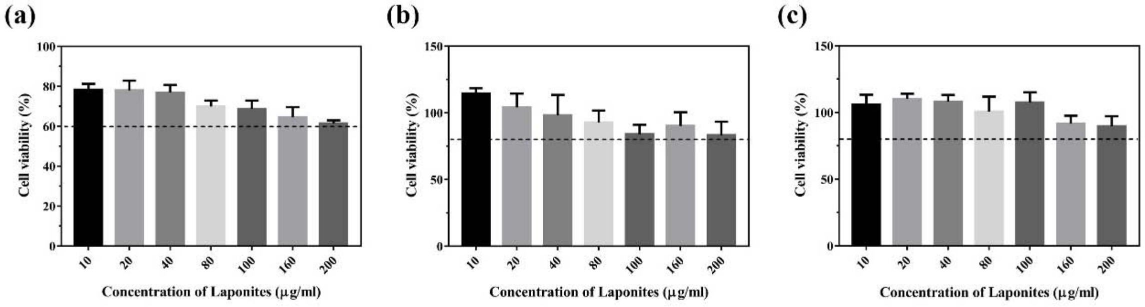

Supplementary Information Figure S6. Cytotoxicity assays of three sized hectorites. The cytotoxicity of various hectorites including (a) LRD, (b) HEC and (c) LFN against Hela cell at different concentration was evaluated by MTT assay after 24 hour incubation. Data are expressed as mean ± S.D. (n=6).

Cytotoxicity assays: HeLa cells were cultured with DMEM containing 10% FBS at 37 °C under 5% CO_2 atmosphere. Then cells were seeded in 96 well plate at 5000 per well and culture for one day. After 24 hour incubation with hectorites at various concentrations, the cell viabilities were detected by the 3-(4,5-dimethylthiazol-2-yl)-2,5-diphenyltetrazolium bromide (MTT) assay. Briefly, 10 μL of 5 mg/mL MTT was added into each well, incubating for 4 hours in dark. Subsequently, supernatants were replaced by 100 μL of DMSO for dissolution of the MTT formazan. Results (n=6) were obtained via measurements of absorbance at 570 nm while the viability was further calculated as following equation:

$$\text{Cell viability (\%)} = (A_m/A_c) \times 100\%$$

The A_m stands for the values measured, while the A_c means the control group without any nanoparticle treatment but with the same culture conditions.

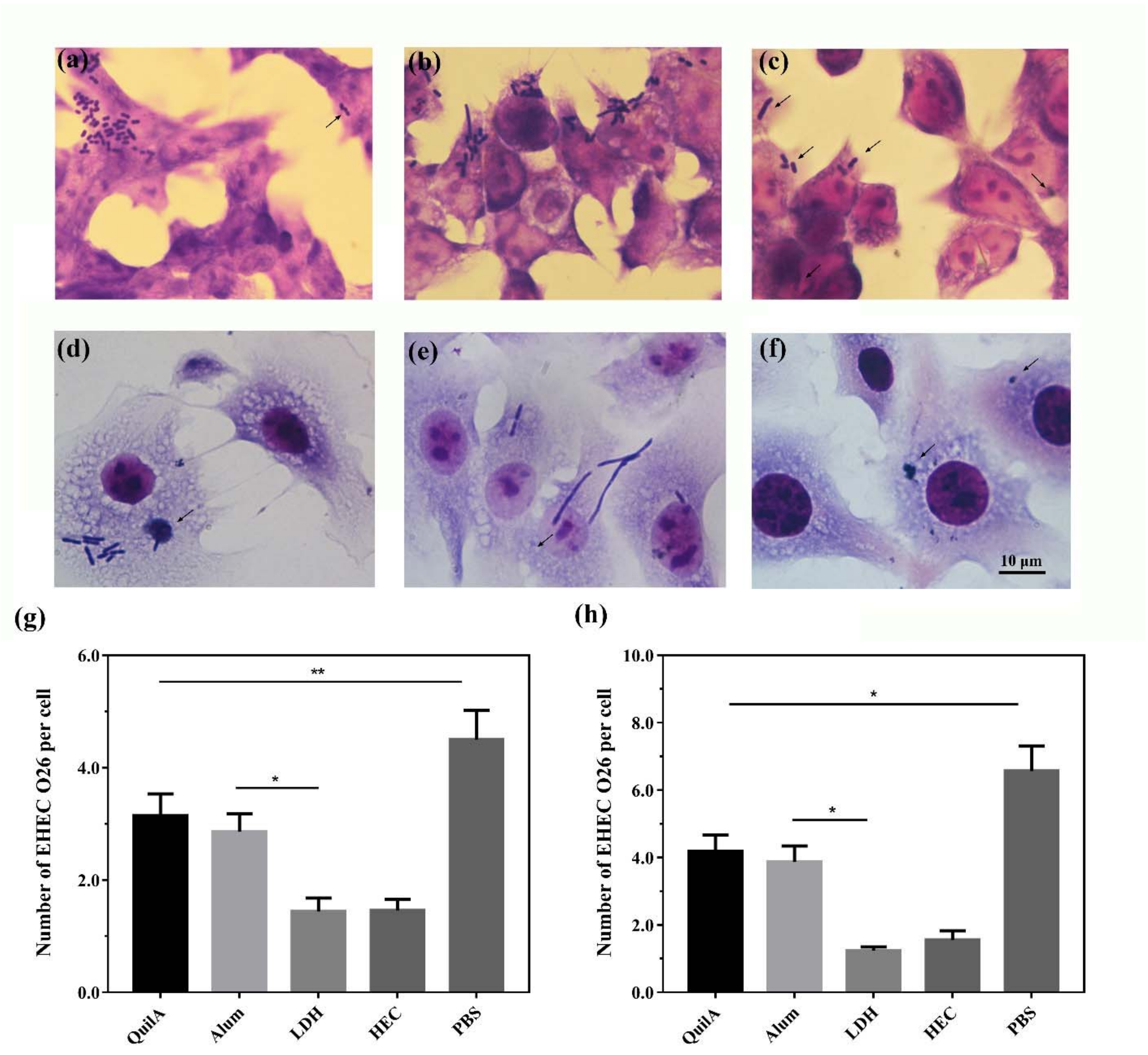

Supplementary Information Figure S7. Blockade attachment of EHEC O26 triggered by SIgA extracted from Nanoparticles vaccine groups in (**a-c**) **MDBK and** (**d-f**) **HRT-18 cell line**. After GIEMSA stain, Cells treated with fecal-SIgA extractions from (**a,d**) QuilA, (**b,e**) Alum and (**c,f**) LDH. Histograms show the number of bacteria attaching to each (**g**) MDBK or (**h**) HRT-18 cell calculated by morphometric technique. Data are expressed as mean ± S.E.M. (n=4). *, $P < 0.05$; **, $P < 0.01$; ***, $P < 0.001$; and ****, $P<0.0001$. Scale bar = 20 µm. Scale bar = 10 µm.

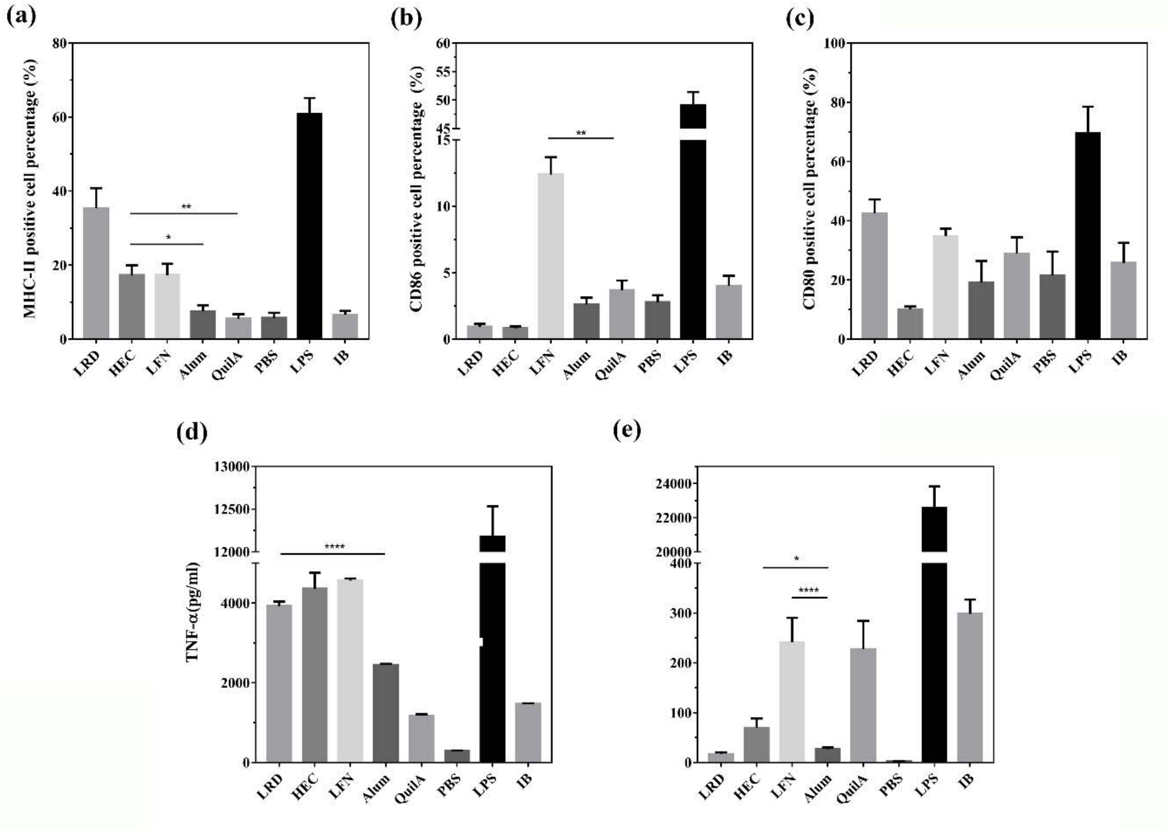

Supplementary Information Figure S8. Stimulation of macrophage induced by clay nanoparticles combined with IB. Changes of surface markers from RAW 264.7 cells were determined after stimulation by clay nanoparticles (with mass ratio of nanoparticles to IB at 32:1) or commercial adjuvants (with mass ratio of Alum and Quila to IB at 32:1 and 1:1 respectively) with combination of antigen IB (with final concentration at 0.5 μg/mL). PBS, IB (0.5 μg/mL) and LPS (100 ng/mL) were used as blank, negative and positive control. Positive percentage of (**a**) MHC-II, (**b**) CD86 and (**c**) CD80 on cell surface were examine by FACS. Cytokines, (**d**) TNF-α and (**e**) IL-6 secreted in supernatant followed excitation were also assayed via ELISA. Data are expressed as mean ± S.E.M. (n=4). *, $P < 0.05$; **, $P < 0.01$; ***, $P < 0.001$; and ****, $P<0.0001$.

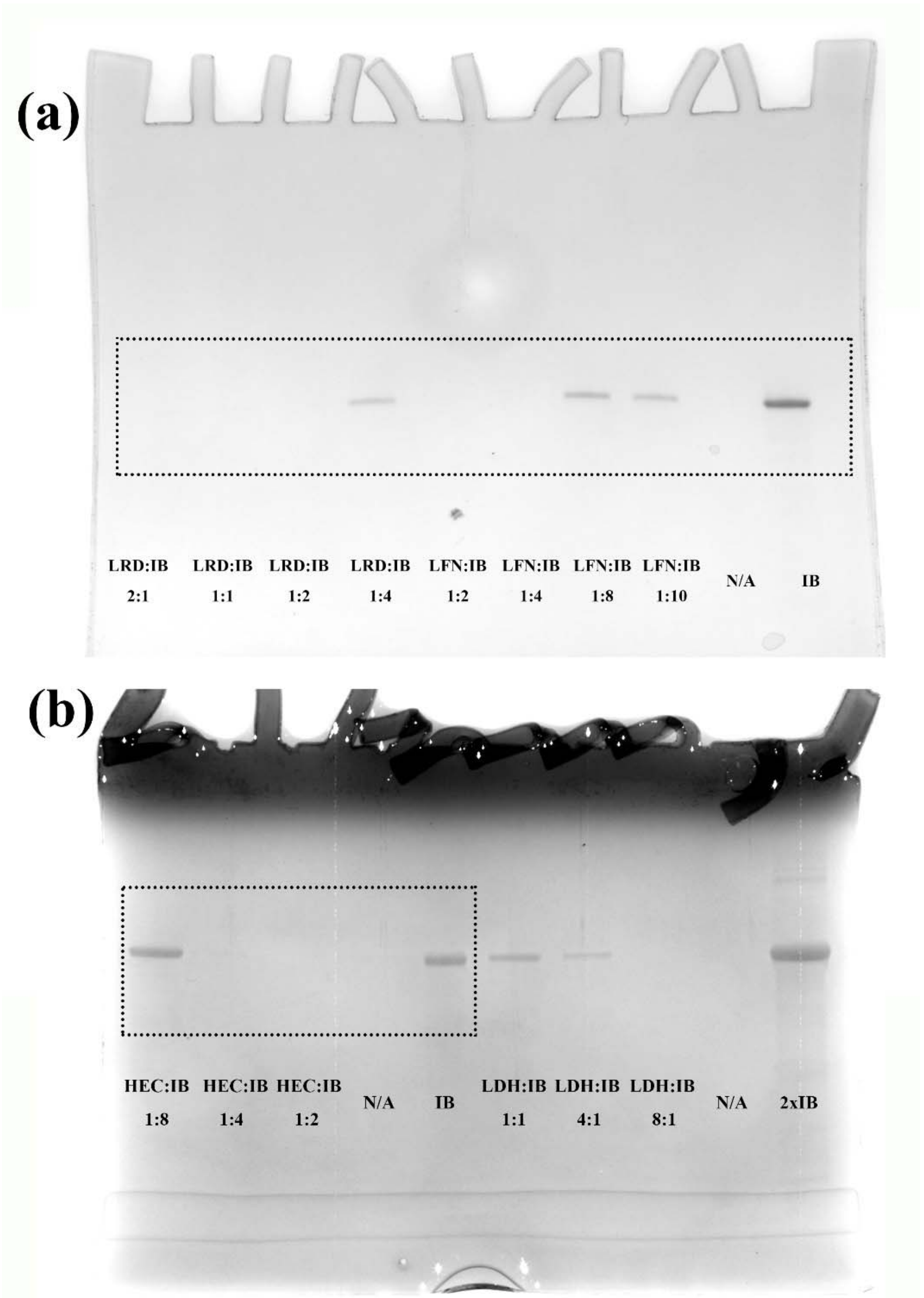

Supplementary Information Figure S9. SDS-Page of free IB in supernatant after binding with NPs. The free IB in supernatants of different binding mass ratios of (a) LRD/LFN or (b) HEC to IB were examined by SDS-PAGE. The frames with dash line indicate the cropped areas presented in Figure 2.
